# Health outcomes following COVID-19 infection and vaccination in hereditary hemorrhagic telangiectasia

**DOI:** 10.1186/s13023-025-03561-2

**Published:** 2025-03-01

**Authors:** Christopher M. Tarulli, Xiayi Ma, Kamalprit Chokar, Nicholas T. Vozoris, Marianne S. Clancy, Marie E. Faughnan

**Affiliations:** 1 Toronto HHT Centre, Division of Respirology, Department of Medicine, St. Michael’s Hospital and Li Ka Shing Knowledge Institute, University of Toronto, 30 Bond Street, Toronto, ON M5B 1W8 Canada; 2 Cure HHT, Monkton, MD USA

**Keywords:** HHT, Vaccination, COVID

## Abstract

**Background:**

There has been concern that individuals living with Hereditary Hemorrhagic Telangiectasia (HHT) could be at higher risk for poor outcomes if infected with SARS-CoV2, the virus that causes COVID-19 disease. As literature is lacking on outcomes on COVID-19 infection and vaccination in HHT, the objectives of this study were to determine and assess outcomes in HHT, as well as quantify vaccination rates and vaccination side effects in a large cohort of individuals with HHT.

**Method:**

Individuals previously recruited to OUR HHT Registry at St. Michael’s Hospital, Toronto were contacted for participation in this study. Data were collected during annual assessment through a series of questionnaires asking specifically about HHT complications, treatments, and symptom management, along with COVID infection and vaccination data.

**Results:**

We attempted to contact all 262 subjects recruited to the registry. Of these, 215 (82.1%) responded at least once regarding COVID-19 related inquiries between April 2020 and August 2022, and these individuals formed our study sample. Forty-nine COVID-19 infections were reported in 47/215 (21.9%) individuals. Among 47 patients with recorded COVID-19 infection, 2/47 (4.3%) required urgent care and 7/47 (14.9%) were hospitalized following infection. Of the 7 individuals who were hospitalized, 3 (42.9%) required new supplemental oxygen. Zero deaths were reported due to COVID-19 infection. COVID vaccination history was available in 147/215 (68.4%). Of these, 135/147 (91.8%) of individuals reported vaccination and side effects were mild.

**Discussion:**

While our sample population is much like the general HHT population with regards to gender, HHT symptoms, and genetics, study limitations including survivor bias, lack of vaccine effectiveness assessment, and participant reported data should be acknowledged.

**Conclusion:**

Our results suggest that HHT patients are not at higher risk of severe infection with COVID-19 compared to the general population. Vaccination rates are high with only mild side effects being observed.

## Background

Hereditary Hemorrhagic Telangiectasia (HHT), also known as Osler-Weber-Rendu disease, is an autosomal dominant genetic condition that results in abnormal connections between arterial and venous blood vessels called vascular malformations (VMs). It is a multi-system disease that affects the nose, respiratory system, gastrointestinal tract, brain and liver, with current international guidelines for diagnosis and management of disease [1]. Individuals with HHT are primarily at risk for bleeding with 90% of adults suffering from chronic epistaxis, and some authors have suggested increased risk of venous thromboembolism (VTE) in HHT [2].

In March of 2020, a global pandemic was declared due to SARS-Cov-2, the virus that causes COVID-19 disease. The impact of COVID-19 in the HHT population was unknown and there was concern that HHT patients might be at higher risk of complications from COVID-19. Symptoms of COVID-19 infection can result in airway inflammation which could worsen epistaxis, a common complication experienced by individuals with HHT. COVID-19 infection can lead to thrombosis, with abnormal coagulation tests occurring in approximately 20–30% of hospitalized patients [3]. As such, there was concern about increased thrombotic complications in infected HHT patients, as well as the risk of increased bleeding should therapeutic or prophylactic anticoagulation be required. In addition, given the reported cardiac complications associated with COVID-19 vaccination, although very rare, there was concern that this could negatively impact patients with HHT-related cardiomyopathy. Vaccination against COVID-19 can reduce risk of infection and minimize symptoms once infection occurs, and so more information is needed to balance the risk analysis in HHT.

Health outcomes following COVID-19 infection in the HHT population are lacking and there is little published literature on vaccine uptake in this population, particularly because HHT is a rare disease and there are limited population-based studies in this area. One study conducted early-on in the pandemic in Italy examined participants in an HHT registry and reported COVID-19 infection only in 9/296 (3%). Of the 9 infected, 2 were hospitalized and no deaths were reported [4]. A cohort of Spanish HHT patients observed COVID-19 infection in 25/138 (18%), and only 3/25 (12%) required hospitalization, and there were no deaths [5]. Lastly, a case study regarding an elderly woman with HHT who contracted COVID-19 and died shortly after [6], along with two letters to the editor [7, 8] further highlight the importance of deeper investigation on this topic.

The objectives of this study were (1) to describe the symptoms and clinical course of COVID-19 infection in individuals with HHT and (2) to assess vaccine uptake and side effects among a large cohort of HHT patients. This study will add to the existing literature and help HHT patients with informed decision making.

## Methods

This study was approved by the local Research Ethics Board. Individuals enrolled in OUR HHT Registry from the Toronto HHT Centre at St. Michael’s Hospital were included in this study. OUR HHT Registry is a longitudinal outcomes registry of people with definite HHT. Between April 2020 and August 2022, during routine annual follow up for OUR HHT Registry, participant-reported data were collected regarding COVID-19 infection (symptoms, treatment and outcomes, including hospitalization and vital status) and vaccination (events and side effects), in addition to HHT symptoms, treatments, and outcomes. Collection of vaccination data began July 2nd, 2021, after vaccinations readily available to the public. Data collection ceased in August 2022, when study funding ended. Vaccination side effects were classified as flu-like symptoms, injection site symptoms, thrombosis complications, allergic type symptoms, hospitalization and vital status. Data were collected via questionnaires, administered over the phone or during in-person routine clinic visits.

### Statistical analysis

Data were summarized using mean +/- standard deviation for continuous variables and frequency and proportion for categorical variables. Analyses were done using the open-source software R version 4.3.0.

## Results

**Fig. 1:**
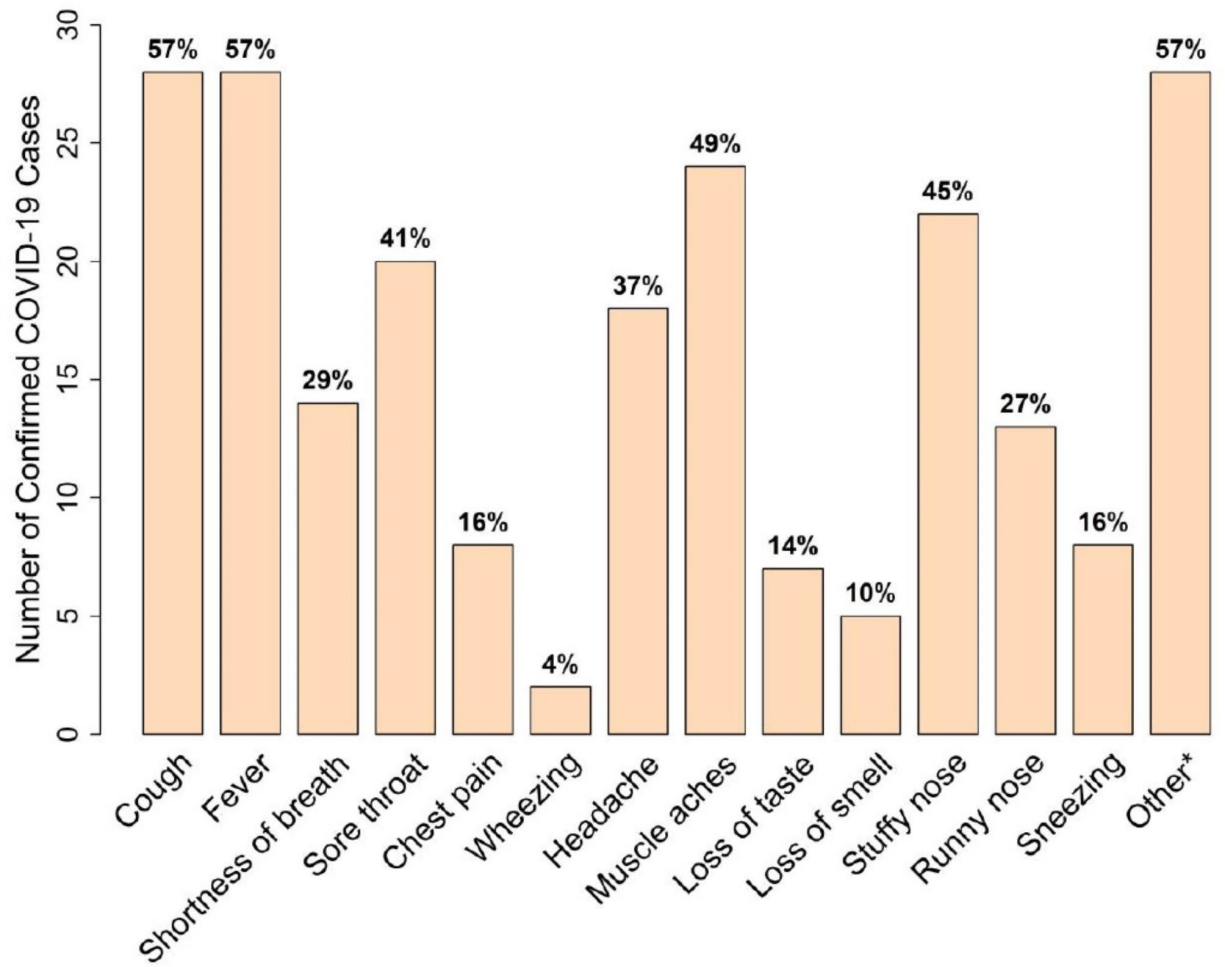
Number of COVID-19 cases with typical symptoms (Percentage of 49 infections shown). *Common other symptoms included armpit swelling/soreness, and itchiness in various locations

Our study population consisted of the 262 participants recruited from Toronto HHT Centre at St. Michael’s Hospital to OUR HHT Registry. The characteristics of these individuals are summarized in Table 1. We attempted to contact all 262 individuals recruited to the registry. Of these, 215 (82.1%) responded at least once regarding COVID-19 related inquiries between April 2020 and August 2022, and these individuals formed our study sample. Out of our study sample, 47 (21.9%) reported COVID-19 infection, with two individuals reporting a second COVID-19 infection. Symptoms following COVID-19 are shown in Fig. 1, with cough and fever being the two most commonly described symptoms (both were reported by 57.1%). Among 47 patients with recorded COVID-19 infection, 2 (4.3%) required urgent care and 7 (14.9%) were hospitalized following infection. Out of the 7 individuals who were hospitalized, 3 (42.8%) required supplemental oxygen. Worsening epistaxis was reported in 4/47 (8.5%) individuals with COVID-19. The infections occurred mostly during the Omicron variant wave 34/49 (69.4%), from November 1 2021– June 1 2022. There were no deaths.

**Table 1:** Population characteristics of 213 HHT patients contacted from the Toronto HHT Centre to OUR HHT Registry

Characteristic	Proportion Affected	Percent Affected
**Female**	126/213	59.2
**Male**	87/213	40.8
**ENG**	88/188	46.8
**ACVRL1**	83/188	44.1
**SMAD4**	7/188	3.7
**Negative for all 3**	10/188	5.3
**Brain Arteriovenous Malformations**	18/209	8.6
**Pulmonary Arteriovenous Malformations**	96/212	45.3
**Liver Vascular Malformations**	53/130	40.8
**Epistaxis**	205/213	96.2
**GI Bleeding**	70/211	33.2
**Mucocutaneous Telangiectasias**	201/213	94.4

**Fig. 2:**
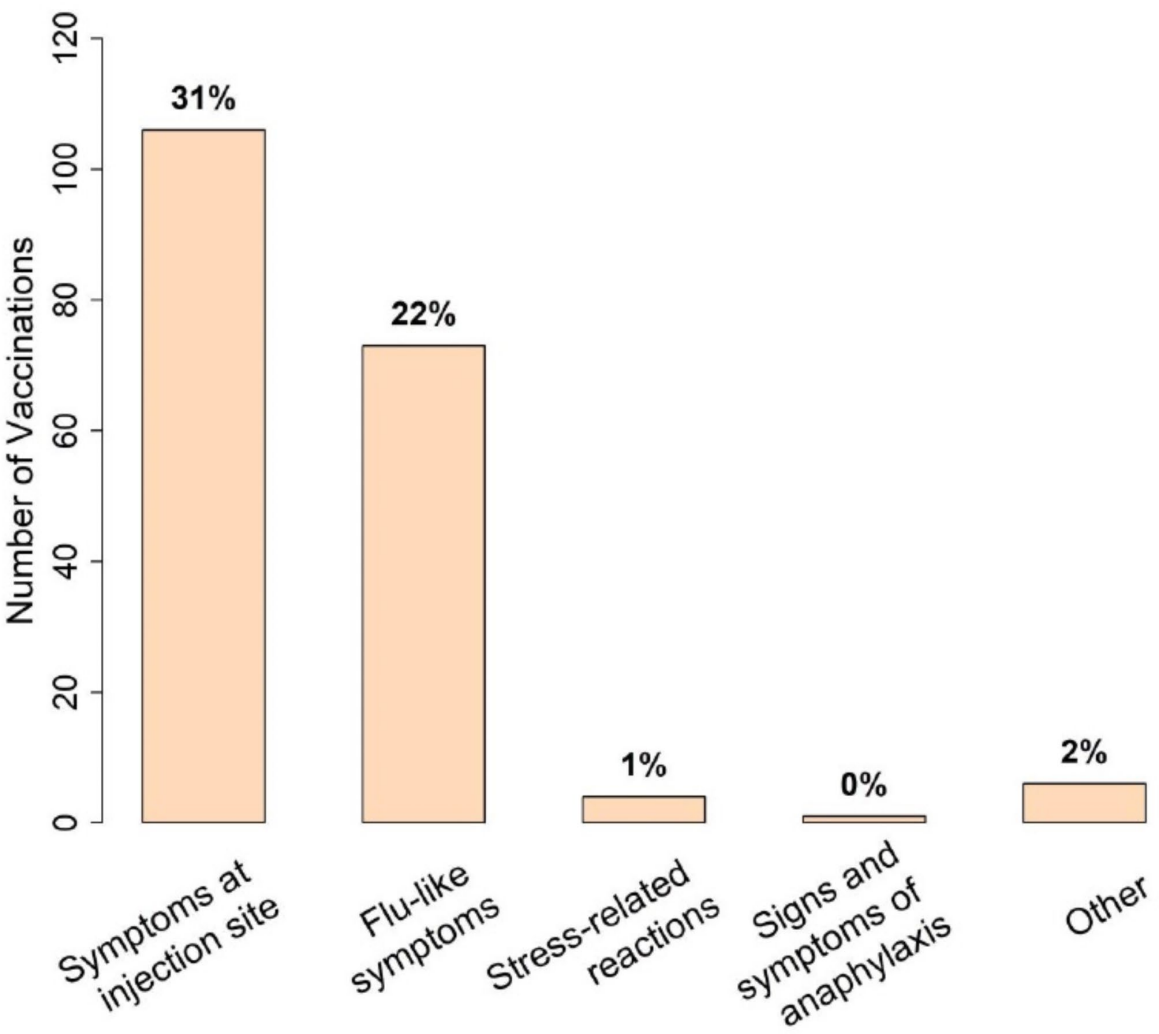
Number of vaccinations with typical side effects were reported (Percentage of 339 vaccinations shown)

We attempted to contact all 215 subjects within our study sample once vaccinations were approved and available. Of these, 147 (68.3%) responded and provided vaccination data. 135/147 (91.8%) reported receiving at least one vaccination, with most receiving at least two doses. Of 339 total vaccination doses, 138/339 (40.7%) reported side effects. A break-down of vaccination side-effect categorization is outlined in Fig. 2, with local injection site symptoms being the commonly reported vaccine side-effect (31.3%), followed by flu-like reaction (21.5%). VTE complicated 7/339 (2.0%) vaccination doses.

## Discussion

Our study describes the largest case series of COVID-19 infections in persons with HHT and is one of the first to report on vaccination in this population. The HHT population recruited to our Toronto HHT Registry is similar to the general HHT population with regards to gender, HHT symptoms, and genetics^5^ [9]. Overall, COVID-19 in our HHT population does not appear to frequently result in severe disease outcomes, nor in epistaxis escalation. In addition, vaccine uptake was high in our HHT cohort and was associated with mostly mild side effects. Since it appears that in the foreseeable future, COVID-19 will likely continue to circulate to some degree world-wide, the number of affected HHT patients will undoubtedly progressively rise, and it is reassuring to observe that COVID-19 infection outcomes and vaccination side-effects are similar among individuals with HHT and the general population.

Several study limitations need to be acknowledged. First, our study did not evaluate the effectiveness of COVID-19 vaccination in the HHT population. Second, individuals with HHT that died of COVID-19, either before recruitment to OUR HHT Registry OR before COVID-19 was routinely diagnosed, may have resulted in survivor bias. Third, our study data was all participant-reported (and not objectively confirmed), and therefore, subject to recall and social desirability biases. Fourth, approximately 20% of individuals in OUR HHT Registry did not participant in our study. Some of our findings may be different had data on these individuals been included, although the characteristics of our study sample were similar compared to individuals in the OUR HHT Registry. A fifth limitation is that our study is confined to the highly developed healthcare system in Toronto, and observed outcomes may not be generalizable to regions where healthcare infrastructure is underdeveloped or was overwhelmed, particularly in surges of COVID-19 cases.

Similar to the few other studies describing COVD-19 infection amongst persons with HHT [4, 5] we reported that relatively low numbers of patients (only 23%) contracted COVID-19. Several factors could explain the relatively low frequency of COVID-19 in our HHT patient study sample. First, our study data collection occurred mainly during the first year of the pandemic and does not reflect the higher cumulative frequency reported over time. We suspect that individuals with HHT may have been strongly motivated to follow recommended precautions as result of having HHT and uncertain risks if infected with COVID-19, particularly early in the pandemic, which may have served to protect individuals from acquiring infection. During the pandemic, the use of online appointments and virtual care when possible likely served to reduce the need for in-person care, and as result, risk of exposure to COVID-19 in the hospital or clinic setting. Another factor that could have influenced the relatively low COVID-19 detection rate is individuals with HHT may have been reluctant to undergo nasopharyngeal swab testing because of the fear of precipitating an episode of epistaxis. Though oropharyngeal swabs were recommended, their availability in the community was variable.

Another important factor to highlight is that epistaxis was reported to worsen during COVID-19 infection in only 8.5% of individuals, which is surprisingly low as COVID-19 is known to affect the nasopharyngeal space. This could be due to the relatively mild symptoms that most individuals experience with COVID-19, including our HHT population.

Vaccine-associated VTE was reported infrequently, but more frequently than in the general population, with 2% of vaccinations being complicated by VTE compared to less than 0.1% in the general population^6^ [10]. This is most likely due to the small sample size or alternatively could be due to possible increased risk of VTE in HHT patients. Lastly, individuals with known COVID-19 infections were purposely sought after for recruitment to OUR HHT registry, although prevalence of COVID-19 was still low in our sample population.

## Conclusion

Ultimately, individuals with HHT do not appear to be more susceptible to severe COVID-19 complications than the general population. HHT patients do not appear to be at higher risk of complications related to COVID-19 vaccinations. The number of HHT patients acquiring HHT will undoubtedly continue to rise, and it is reassuring to observe that COVID-19 infection and vaccination outcomes in HHT are similar to the general population.

## Data Availability

The datasets generated and/or analyzed during the current study are not publicly available due protection of privacy but are available from the corresponding author on reasonable request.
